# Limits to human enhancement: nature, disease, therapy or betterment?

**DOI:** 10.1186/s12910-017-0215-8

**Published:** 2017-10-10

**Authors:** Bjørn Hofmann

**Affiliations:** 1 Norwegian University of Science and Technology (NTNU), Gjøvik, Norway; 2 Centre for Medical Ethics, University of Oslo, Blindern, PO Box 1130, N-0318 Oslo, Norway

**Keywords:** Enhancement, Disease, Therapy, Naturalness, Nature, Limits

## Abstract

**Background:**

New technologies facilitate the enhancement of a wide range of human dispositions, capacities, or abilities. While it is argued that we need to set limits to human enhancement, it is unclear where we should find resources to set such limits.

**Discussion:**

Traditional routes for setting limits, such as referring to nature, the therapy-enhancement distinction, and the health-disease distinction, turn out to have some shortcomings. However, upon closer scrutiny the concept of enhancement is based on vague conceptions of what is to be enhanced. Explaining why it is better to become older, stronger, and more intelligent presupposes a clear conception of goodness, which is seldom provided. In particular, the qualitative *better* is frequently confused with the quantitative *more*. We may therefore not need “external” measures for setting its limits – they are available in the concept of enhancement itself.

**Summary:**

While there may be shortcomings in traditional sources of limit setting to human enhancement, such as nature, therapy, and disease, such approaches may not be necessary. The specification-of-betterment problem inherent in the conception of human enhancement itself provides means to restrict its unwarranted proliferation. We only need to demand clear, sustainable, obtainable goals for enhancement that are based on evidence, and not on lofty speculations, hypes, analogies, or weak associations. Human enhancements that specify what will become better, and provide adequate evidence, are good and should be pursued. Others should not be accepted.

“*Don't know what I want / But I know how to get it.*”Johnny Rotten

## Background

Human enhancement (HE) is defined as “any kind of genetic, biomedical, or pharmaceutical intervention aimed at improving human dispositions, capacities, or well-being, even if there is not pathology to be treated” [1]. The topic has stirred vast and vivid debates, especially in the bioethics literature [2–6].

There seem to be two predominant camps in the debate on HE: a *permissive* and a *prohibitive* [1]. While the first camp sees few limitations to HE, the latter sees many. While the permissive position provides arguments from potential benefits, the prohibitive position refers to (religious and nonreligious) conceptions of sanctity, dignity, and on the notion of “playing God.” In addition, there is a *restrictive* camp [1], not being in principle against enhancement, but which argues for limits from concerns for justice and safety. However, there are few specifications of how to limit HE in detail.

In this article I will investigate four specific routes to limiting HE: Naturalness, disease, therapy, and betterment. The corresponding research questions are:

1. Do conceptions of naturalness provide resources to set limits to HE?
2. Does the therapy-enhancement distinction provide resources for setting such limits?
3. Does the concept of disease offer sources to limit HE?
4. Does the concept of HE itself contain means for setting limits?

While I will show that the first three questions do not provide convincing answers, the latter may. However, before I address these questions successively, I must address the following question.

### Why should there be limits to human enhancement?

There appear to be many motivations and arguments for why we should try to limit human enhancement. Allow me to mention a few of these:

- **Resources**: HE has significant *opportunity cost* and would drain resources from more pressing needs. This of course relates to:
- **Justice**: There are not enough resources available for human enhancement for all, and to focus on human enhancement under such conditions can enhance disparity and injustice.
- **Humanity**: Enhancement will change not only the human condition, but humanity as such. One may lose something charitable without knowing what will follow.
- **Uncertainty** about benefits and harms: HE may have unknown and potentially devastating consequences.

In this work I take it as a premise that HE needs to be limited for some valid reason. At the end of the article, I will return to this premise. Although the traditional approaches to set limits to enhancement may not be convincing, they may not have to do such heavy work. The limits to enhancement may be found in the conception of enhancement itself. Investigating the weakness of the arguments from naturalness, the therapy-enhancement distinction, and the concept of disease, may provide assets for exploring the self-limiting potential of human enhancement.

## Discussion

### Naturalness: limits in nature

Several objections to HE and arguments for its limitation are made with reference to human nature as a norm [7]. The natural world has frequently been associated with order and design (*Harmonia Mundi*), either teleological, mechanical, or cybernetic [8–10]. Correspondingly, nature has been normative in many ways, e.g., epistemically, aesthetically, and morally. For example, our knowledge of how to heal a broken leg, how to restore its appearance, and the impetus to do so is drawn from the norm of a well-functioning leg of a being in its natural habitat.

An explicit expression of the norm of nature can be found in an editorial in JAMA from 1928: “The statements that old age can be deferred have no more scientific truth in them than the widely advertised promises of rejuvenation. Senescence is a normal process of involution as necessary to the progress of life as is the normal process of growth. It is intrinsic, inheritable, fixed in the germ plasm through the action of all of the forces of the Universe.” (Quoted from [11]). Most cultures employ a distinction between “nature” and “culture”; “man-made” and “nature-made” [12]. Accordingly, a common understanding of medicine is that it works according to the norms of nature, as it restores a given harmony.

Hence, nature may provide a prima facie norm for setting limits to human enhancement. Let me briefly recapitulate some of these arguments.

#### Arguments from naturalness for limiting human enhancement

One argument why society should not permanently enhance humans is that this would alter our common understanding of human excellence and flourishing in ways that would undermine our social practices [13]. For example, human enhancement would make us lose sight “of why excellence is worth seeking at all, and hence what excellence really is, and how we pursue it as human beings, not as artifacts” [4]. In particular, it would alter the meaning of sports, as we would come to honor technological innovation and not talent and athletic achievement [14].

Another argument to limit enhancement is that human nature is a complex reality and that enhancing one aspect of it could undermine the excellence of the whole [13]. Accordingly, enhancing separate abilities would “disrupt either the unity or the continuity of human nature” [15] and compromise humanity [4], e.g., by diminishing our humanity by decreasing our appreciation for beauty, benevolence, and vulnerability [16]. A related concern is that human enhancement would disturb a delicate balance in nature resulting in unintended and potentially disastrous consequences [17]. Alternatively, we obtain our enhancement goal, but it appears to be counterproductive or undermining something cherished [18, 19].

Yet another concern is that human enhancement would make individuals stand outside the human species, as “post-humans” [13]. Or that human enhancement would violate some special feature of human nature, such as basic dignity. As expressed by Eric Cohen: “All members of the human family – all living bodies – have a human life, and therefore deserve the respect that such membership commands” [20].

Further references to nature is made in claims “that handicap and limitations are inherent to human condition, and should be endured as given by our own individual fates” [7]. Such arguments sometimes are related to human responsibility, i.e., that altering human nature would give us a responsibility that we are not suited to handle, and sometimes related to the (religious or non-religious) metaphor of “playing God” [1, 6].

The point here is not to review all arguments from human nature, but only to give a brief overview and a backdrop for the counterarguments. Moreover, the arguments are not always clear as to whether they address altering humans, human nature, or nature in general. Neither are the counterarguments. Nonetheless, the core issue is that nature, of which human beings are an integrated part, provides some standard for the human being, in terms of abilities, activities, aspirations, and norms for treatment and restoration - and for setting limits to enhancements.

#### Some counterarguments against the naturalness argument

A wide range of counterarguments have been presented against these naturalness-based arguments for limiting (or ban) HE. One much referred counterargument is that human nature is normatively bidirectional, i.e., that it can be both good and bad [21]. Evolution does not guarantee goodness. “[T]he fact that natural selection has operated on a trait does not ensure that the trait is optimal” [5]. Evolution can be improved [22, 23], and human nature may very well include culture and tradition. The assumption that natural things and actions are good, and that artificial things and actions are not good is false and unwarranted [8, 24]. The core of this line of argument is neatly summarized by Lewens in his claim that nature “either constitutes an irrelevant preamble to the important question of which features of human reproduction should be preserved, or it constitutes an objectionable allusion to some mythical and morally loaded ‘human nature’ that might serve as an ethical yardstick in debates of this sort” [21].

On the contrary, it is argued, there can be a moral imperative to change, improve, or reframe human nature, using various forms of technology in the pursuit of some other perfection or ideal [5]. To enhance ourselves “is not eugenic but expresses our fundamental human nature: to make rational decisions and to try to improve ourselves. To be human is to strive to be better” [25]. Hence it is in our human nature to enhance ourselves [26, 27] and to use anthropotechnology to alter and ameliorate ourselves [28].

This line of thought has a range of sources. It can be found in Pico de la Mirandola and Nietzsche claiming the glory of humankind lies within an absence of a fixed nature [8], and it can be traced back to Greek mythology where humans are born without properties, stealing them from others like Prometheus. It is by shaping nature we form ourselves: our human nature. These thoughts may also be connected to conceptions of nature as something to overcome or to a duty to self-civilize with links back to Erasmus and Plato [29]. Or in a Marxian remolding of nature and man into a new social man [30].

Additionally it is argued that human nature is changing, and thus cannot provide a stable conception of what is good. Due to mutations, there is no stable species-typical genome to refer to as natural [31]. Humans do not have natures no more than biological species [21]. Even more, humans have changed their physical, biological, and microbiological nature extensively. What we call “natural” means nothing more than “usual,” which frequently changes: “For example, women naturally menstruate and go through menopause in a way that women do not naturally have in vitro fertilization, tummy tucks, or breast augmentation or reduction. ‘Natural’ in this context means little more than if not tinkered with, women usually do this” [32]. Accordingly, if we were not to tamper with nature, it would bring us back before the Stone Age. Although some would argue that we have tampered enough [33], we “live in a world permeated by natural-artificial hybrids of myriad varieties and stripes, and we may as well make our peace with this ineluctable fact” [34]. As argued by Barilan and Weintraub “[d]ividing the world between nature and culture is artificial. This division is a cultural construct by itself” [8].

Yet another line of counterargument points to the empirically supported fact that there are many conceptions of “nature” in the argument from nature to limit HE. Let me therefore briefly review some notions of nature and natural.

#### Many conceptions of nature and natural in the enhancement debate

There are several conceptions of nature in play in the HE debate [8]. Bess illustrates how human nature is conceived of as an essential core, a blank slate, and as a work-in-progress, and argues that none of them provide solid ground for delimitation of enhancement [34].

Moreover, the Nuffield Council in a recent report has identified five different conceptions of naturalness in public and political debates about science, technology, and medicine:

1. *Neutral*: a neutral/skeptical view that does not equate naturalness with goodness.
2. *Wisdom of nature*: the idea that nature has found the correct or best ways of doing things and should not be “tampered” with.
3. *Natural purpose*: the idea that living things have natural purpose, essence or functions which is linked to what is good for them and which science shouldn’t seek to change.
4. *Disgust and monstrosity*: a response of disgust, revulsion or fear prompted by novel technologies.
5. *God and religion*: the idea that certain technologies distort God’s creation or go against the will of God [35].

The four first conceptions of naturalness have been at the front of the bioethics debates briefly reviewed above. Furthermore, it has been shown that standard philosophical conceptions of human-nature relationships, such as Master, Steward, Partner, and Participant have resonance in people, but that people see themselves as “part of nature” and “responsible for nature” at the same time [36]. This combined conception of naturalness is not well covered in the bioethics debate.

#### Limits to the HE in nature debate and future directions

Although it is widely concluded that nature does not provide norms for limiting HE, there are still conceptions of nature and naturalness that are not well addressed. For instance, Rousseau’s conception of human nature as something we need to rediscover has gained little attention in the bioethical debates. Heidegger’s (and Stiegler’s) conception of humans being technological creatures, at the same time at home and not at home within ourselves and with technology may also provide relevant perspectives on nature and technology facilitating critical reflection on HE. So may Kierkegaards conception of human nature as relation to ourselves, mediated to our pre-connection to the other as well as Spinoza’s distinction between *natura naturans* (in process) and *natura naturata* (as fixed given). Arne Næss’ conception of human nature as deeply connected to the nature of our surroundings may do the same.

The point here is not to argue that these perspectives provide means to set limits to HE. It is rather to indicate that there are alternative conceptions of (human) nature that have not been addressed in the literature, and that may be worth pursuing. Anyhow, no knock down argument has been provided that the conception of nature sets limits to HE, i.e., disagreement still exists and seems warranted. Let me therefore move to the next candidate: the therapy-enhancement distinction.

### Therapy versus enhancement

HE goes beyond medicine as a healing enterprise, or as expressed by Edmund Pellegrino: it “goes beyond the ends of medicine as they traditionally have been held” (Pellegrino 2004) as quoted in [37].

Coenen and co-workers make a fruitful distinction between restorative or preventive, non-enhancing interventions (*restitutio ad integrum*), therapeutic enhancements that allow a patient to perform better than before their disease or accident, and non-therapeutic enhancements which improve natural human abilities or to create new abilities [38]. It is the latter where limits to HE have been most heatedly debated.

One line of argument focuses on the *use* of the technology, and not the technology as such. Technology used for treatment, rehabilitation, and restoration is acceptable, but not for going beyond this. Therapy is usually defined as the use of medical means to restore or establish normal functioning of an organism [4, 39]. Accordingly, enhancement is to go beyond this warranted basic provision of help [40], and the motives are entirely different [41], providing a prima facie argument in favor of limiting enhancement.

The therapy-enhancement distinction is also supported by arguments in professional ethics, where medicine is defined in recuperative terms. Medicine is about treatment, not enhancement [42], and is defined as “a restorative practice aimed at the return to health” [43]. Using technology beyond this goal is to breach with its professional ethos. As clearly expressed by Benditt: “Medicine has always been about healing. The role of physicians is not a matter of what they may have the knowledge and skills to do, but about what constitutes healing. Enhancing, though, is not healing. Therefore it is not within the purview of medicine” [44].

Erik Malmquist supports the distinction between therapy and enhancement (by showing that the continuum-argument fails on three grounds) [45]. First, curing and preventing disease is more fundamental to expand people’s capacity to realize flourishing lives than enhancement. Disease makes people incapacitated and unable to pursue their happiness, and reduces autonomy in other ways. Second, Malmquist points out that disease avoidance can be more accurately specified than enhancement. To remove the preconditions of Tay-Sachs or cystic fibrosis is much easier specified than the preconditions for enhancing traits, such as intelligence. Third, disease avoidance tends to be more urgent than enhancement from the point of view of distributive justice. Malmquist admits that these aspects may not provide a knock down argument for a firm therapy-enhancement distinction, but contends that they show that a continuum model is not an attractive alternative [45].

A sophisticated way to discriminate therapy and enhancement has been based on distinguishing between comparative and non-comparative harms. Not providing dietary supplements during pregnancy (as a treatment) that could prevent the resulting child from developing dyslexia would result in *counterfactual comparative harms* [46] while not enhancing the (cognitive) capacities of the embryo would involve non-comparative harms, as it would not be the same child that would come into existence.

According to these arguments the therapy-enhancement distinction provides resources for setting limits to enhancement. Let me briefly review some of the counterarguments to see if this holds.

#### No sharp therapy-enhancement distinction

A frequently referred (pragmatic) counterargument is that the therapy-enhancement distinction has long been surpassed in “standard-contemporary-medicine” which includes “preventive medicine, palliative care, obstetrics, sports medicine, plastic surgery, contraceptive devices, fertility treatments, cosmetic dental procedures, and much else” [47]. Accordingly, there is no better reason to limit enhancement than to limit these and other types of non-therapeutic activities.

Another argument against the treatment-enhancement distinction is that it is a *moving target.* For one, therapy is connected to the concepts such as “normality” or “health,” which are “fishy” [48], vague [49], and relative [34, 50]. Variation in the population makes it difficult to define pathology, and therefore also therapy. Second, the concept of therapy is vague. The same intervention (with respect to functioning) which in one case may be therapy may be enhancement in another [47]. We use Ritalin to alter behavior or enhance school achievements. Growth hormones are used to alter and enhance stature, and the limits between them is blurred and socially contingent [51] We also modify well-functioning organs (with bariatric surgery) in order to treat Type II Diabetes (independent of weight loss) [52]. Moreover, what is considered as treatment has changed considerably with time. Some forms of assistive reproduction previously seen as enhancement are now considered to be treatments. This vagueness in therapy is mirrored in the classification of interventions. Vaccination can be seen as a form of prevention, but also as an enhancement of the immune system [47]. To distinguish between laser eye surgery and contact lenses or glasses appears artificial. Moreover, it can be argued (as John Harris does) that even if the distinction between disease and enhancement can be established clearly, it does not have a normative force for enhancement. Enhancement is pursuit on its own merits.

Another counterargument endorses a welfarist conception of medicine where therapy and enhancement have the same goal, i.e., to improve the welfare of human beings [1, 53, 54]. Any technology, be it therapeutic or enhancing, is acceptable as long as it in sum improves human welfare. So goes the argument. In line with this, Savulescu claims that all “[t]reatments are enhancements. Treatments are a subclass of enhancements” [37]. Correspondingly, it is argued that “[m]edicine is inseparable from human culture and creativity. Even when it acts restoratively, its aim is the restoration of a creative and created life plan and values” [8].

Using cochlear implants as an analogy it can be argued that we are already cyborgs, but also that future enhancements can be resisted (as the deaf community has resisted cochlear implants) and that such enhancements need extra measures in order to respect persons who for various reasons do not want to be enhanced [55].

Another line of argument shows that most reasonable interpretations of the concept of need supports publically funded cognitive enhancement [56] as “normal simply is not good enough anymore” [56]. The treatment imperative is flexible.

In summary, while the arguments for limiting HE from the therapy-enhancement distinction are not convincing, no knock down counter-arguments are provided either. There still is reasonable disagreement. In particular, closer scrutiny of the concept of therapy, e.g., via its etymological core, curing, healing, and attendance, may provide fruitful sources for setting limits to HE. This is beyond the scope of this article. Let me therefore move to the next candidate: the disease-health distinction.

### Disease as a barrier to enhancement

I have elsewhere argued that the concept of health does not provide resources for setting limits to enhancement (Hofmann B. Human enhancement: Enhancing health or harnessing happiness? Submitted). On the contrary, human enhancement is based on and extends traditional conceptions of health. I will therefore here concentrate on the concept of disease, and whether it is suitable to delimit enhancement.

#### Differentiating by disease

The boundaries of medicine are traditionally drawn at curing, preventing, and palliating *disease*. As succinctly expressed by Benditt “medicine is concerned with disease, which may be understood in terms of deviation from the normal, which it is the role of medicine to restore. But, the line of thought frequently continues, it is not the role of medicine to go beyond this, to provide enhancements” [44]. Disease calls for action in ways that health and enhancement does not. As stated by Lawrie Reznek 30 years ago: “Judging that some condition is a disease commits one to stamping it out. And judging that a condition is not a disease commits one to preventing its medical treatment” [57]. Disease is a deviance from the natural condition of health and justifies interventions, which enhancement does not (with the same justification).

#### Differentiating on disease won’t do the trick

A series of arguments have been formed against this traditional distinction between disease and enhancement. One line of argument underscores that the concept of disease alters with time and place [58], is vague or fuzzy [59], difficult to define [60], and useless [61]. Increasingly, what was previously considered to be expected deterioration or “wear of life” is now labelled and treated as disease. As explicitly spelled out by Morrison: “the distinction between being healthy or having a disease (classifications based on the present) becomes blurred as new categories of ‘pre-disease’ create a class of ‘patients in waiting’ who have one or more detectable molecular abnormalities and may or may not go on to develop symptomatic disease (a classification based on the future)” [62]. “Standards of normalcy serve as scales of a second order of diagnosis and monitoring, but they do not define states of health and disease. A ‘normal’ red blood cell count does not make a person ‘normal’. It is a normalcy only within the context of specific clinical questions” [8]. Hence, so goes the argument, the disease-health distinction cannot provide clear limits to enhancement.

As already alluded to, the welfarist conception of medicine also undermines the distinction between health and disease. According to Schermer, “it is not self-evident that enhancement falls outside the goals of medicine, if we agree that medicine should aim at well-being, quality of life, or fulfilling vital goals” [42]. In the same wain Savulescu tells us that, “[i]t is not [disease] which is important. People often trade length of life for non-health related well-being. Non-disease [states] may prevent us from leading the best life” [63]. In another work Savulescu argues that “on all concepts of disease, the mutually exclusive distinction between treatment and enhancement is a false one. … Diseases are a subclass of disabilities” [37]. Treating disease or compensating disabilities are just specific forms of human enhancement.

However, a general rejection of disease as delimiting concept of human enhancement may be too hasty and superficial, as there are distinct theories of disease which may offer different means for setting limits to enhancement. Let me therefore briefly investigate three traditional conceptions of disease, i.e., the normativistic, naturalistic, and hybrid conception of disease.

##### Normativistic conceptions of disease

Normativistic conceptions of disease see disease as given by social and cultural norms and not by nature [64]. What falls under the concept of disease is flexible. One strand of normativistic conceptions of disease (and health) can be traced back to Nietzsche’s reflections in The gay science where he claims that: “the more we put aside the dogma of ‘the equality of men’, the more must the … the normal course of an illness be abandoned by our physicians. Only then would the time have come to reflect on the health and sickness of the soul, and to find the peculiar virtue of each man in the health of his soul: in one person’s case this health could, of course, look like the opposite of health in another person” [65]. Hence, the normativistic conception of disease is unsuitable to set limits to enhancement. Anything can in principle become disease and thus the subject to medical attention.

##### Naturalistic conceptions of disease

Naturalistic conceptions of disease are considered to offer more resistance to enhancement. Disease is defined in terms of dysfunction or subnormal functioning [6, 66–69]. To interfere with “species-typical functioning” is therefore to reach beyond the justified sphere of medicine and health care [6] (p.72).

However, removing (and avoiding) disease is conceived of as (a subclass of) enhancing health, especially by welfarists. Nonetheless, the welfarist approach does not bite on naturalists clinging to species-typical functioning [67, 70, 71] as they do not subscribe to its premises.

Against this it can be argued that what is species-typical or normal is not necessarily desirable: a reduced protection of ozone would make white people vulnerable to radiation and skin cancer and “whites might have disabilities relative to blacks even though their functioning was quite species typical or normal” [72]. Others would argue that setting limits to “normal” and providing cut-off values for “natural functioning” involves some evaluation of “the good life,” and hence, of moral values. Various forms of interventions that change the reference class or alter normal functioning of a species [67] also alters disease. To this it can be responded that radiation exposure is not species typical and that thresholds are not related to human values [67, 73].

While offering some substantial resistance for those believing in biological norms, there is still reasonable disagreement on whether naturalistic conceptions of disease provide measures to limit enhancement. Even more, HE can challenge naturalistic conceptions of disease.

##### Hybrid conceptions of disease

Hybrid conceptions of disease see disease as having both naturalistic and normativistic elements. As such that could provide a combination of naturalistic objections with more normative reasons to limit enhancement. However, the normativistic element, such as “harmful”, [74, 75] does not provide powerful limit-setting measures. It can definitely be harmful for a person not to have a high intelligence if the person lives in a context where intelligence is (the only thing) meriting. Moreover, the naturalistic element of hybrid conceptions of disease only bites if you subscribe to a biologic axiology.

Altogether the (naturalistic) conception of disease provides more assets for limiting HE than the naturalness-argument and the therapy-enhancement distinction, especially if you are based in a biological axiology. However, in an anthropocentric axiology, one would argue that we need a morally normative element in order to be action guiding. Biology is not sufficient. Moreover, it can be argued (pragmatically) that so far the concept of disease has not been able to restrict health care activities.^1^ Health care goals go way beyond handling disease already, and thus, will not be suitable to limit human enhancement.

I have now briefly reviewed the traditional sources of setting limits to HE, i.e., naturalness, the therapy-enhancement distinction. Although they still have potentials for providing limits to HE, so far, reasonable disagreements still exist. Hence, they do not provide knock down arguments for limiting HE.

However, this does not mean that there are no means to set limits to HE. Limits to enhancement may be found in the concept of enhancement itself, or more precisely, in the limited power of deducing goodness from enhancement.

### Enhancement and the notion of good

One main challenge with the literature promoting enhancement is that it is unclear what “enhancement” means. As Bess points out, the meaning of enhancement varies along three dimensions:

- Differences of degree: tweaking vs. transmogrifying
- Differences of mode: boosting vs. adding vs. radical remolding
- Differences of relative effect: competitive advantage vs. intrinsic benefit [34].

According to Bess, this makes the use of the term enhancement slippery and controversial. In particular, there tends to be a confusion of *better* with *more* [34].

#### Confusing better with more

One example of this is the argument that it is better for humanity to have people with higher rather than lower IQ scores. “On average, people with a high IQ have better jobs, eat healthier, are less superstitious, and are less likely to be either violent or the victims of violence” [76]. Or here in the words of Dan Brock: “But suppose that a trait such as memory could be genetically enhanced so that everyone could have a “photographic” memory, or that everyone could be brought up to what is now the high end of the normal range of intelligence” [77].

As Bess points out, there seems to be a confusion between the quantitative conception of enhancement, i.e., as *augmentation*, and the evaluative conception, i.e., as *improvement* [34]. However, as pointed out by Parens and others, more is not always better [78, 79]. As with aging, it is far from obvious that living longer is better. Previous reflections on the relationship between more and better seem to be ignored. Titonus obtained eternal life, but found it meaningless. Virginia Wolf’s Orlando lived for 400 years but found it toiling. In Douglas Adams’ Hitchhiker’s Guide to the Galaxy, Wobagger lives eternal, but is miserable.

Michael Hauskeller points out that it is very hard to answer in advance and out of context what is better. The reason for this is that all, or, at least, most traits suggested by enhancement enthusiasts – physical strength, intelligence, emotional stability, long-livedness, predispositions to feel happy, etc. – are complex traits that interact with other traits of a person in unforeseeable ways [80]. It is difficult to determine what a good human being is and which lives are “better” than normal. The reason why it is so difficult to tell what is a good human being, compared to, say, a coffee machine, is that as human beings “we do not have a definite purpose” [80]. Although, thinking is part of human characteristics and it is considered to be bad for human beings to be prevented from thinking, it does not follow that thinking more is better [80].

The apparent tendency amongst the proponents of HE to associate more with better (more strength and endurance, more intelligence, longer lives) seems analogous with the strong cognitive associations between morality and health as well as sinfulness and disease [81] (pp. 208–209). In this, it is argued that ethics seems to go along with the hypes and hopes of emerging technologies far too easily [82–84]. Contrary to the complaint about a “status quo bias” [22], there tends to be a corresponding “change bias.”

Apparently, this problem is acknowledged. As pointed out by Earp and colleagues “more is not always better, and sometimes less is more” [85]. To underscore this they introduce the notion of “diminishment as enhancement.” By defending a welfarist conception of enhancement they believe to have solved the problem. However, to include *less* in the quantitative measure (in addition to *more*) does not provide content to the qualitative conception of *goodness*.

To define goodness in terms of individual persons’ wellbeing may not do the trick either. Savulescu defines human enhancement as “improvement of the person’s life. The improvement is some change in state of the person—biological or psychological—which is good. Which changes are good depends on the value we are seeking to promote or maximize. In the context of human enhancement, the value in question is the goodness of a person’s life, that is, his/her well-being” [37]. There are many reasons why conceiving enhancement in terms of increased wellbeing may not be convincing. First and foremost, what maximizes the wellbeing of the individual may undermine the wellbeing for groups of individuals and for society at large. Relativizing wellbeing to individuals may not make the world a better place. The argument only has traction if you subscribe to a specific philosophical position, which, despite great progress, still is far from all-encompassing. Second, it still is an empirical premise that enhancing certain traits (either by augmentation or attenuation) would in fact increase wellbeing (or other forms of goodness), which is not proven to be true. No doubt, social media have increased the number of social interconnections, but whether it has improved our wellbeing is an open question. With Schramme one can argue: “The improvement of human life can only be considered a collective duty if we know that the results would really constitute an improvement” [70].

Hence, more work is needed to show that something is an enhancement, and explaining enhancement in terms of augmentation (or diminution) appears not to do the trick.

#### Forward in all directions

Accordingly, the argument for HE without a more elaborate conception of betterment (than individual wellbeing) is not convincing, as *better* is different from *more* and *individual wellbeing* is complexly entangled in social involvedness. Defining enhancement in terms of increased individual wellbeing is hardly helpful, as wellbeing appears to be as broad and contestable a concept as health. The argument from change or *argumentum ad novitatem* does not work either. Even if we need to change, it is not obvious that HE is for the good.

To counter this problem it is argued that opponents of HE are subject to a status-quo-bias [22, 23, 86]. As clearly stated by Kahane and Savulescu: “This is, perhaps, the best diagnosis for the status quo bias that infects so many protagonists in the debate – since we don’t know what would be better, we should remain where we are” [23]. However, this critique does not bite because it turns the question on its head. What is asked is rather this: If you argue that we should go forward, you should show that doing so makes life better. If you claim to improve something, then you should give convincing arguments that the change that you suggest actually is better.

Hence, the better clause of HE provides an ingrained measure for limit-setting to HE, or more precicely, to guide human enhancement. When you say we should enhance X because it will make the world better, it is fair to ask why the world becomes a better place from enhancing X. Arguing that enhancement (in terms of change) is better in and of itself is a(n opposite) equivalent to the status-quo-bias, i.e., a “*progress bias*.”

Proponents of HE argue vigorously that there are objective values to show what betterment is. They suggest that “only radical relativist or nihilist could hold that there are no robust values that can guide enhancement. For example, one basic element of morality is willingness in certain situations to make selfsacrificial decisions for the benefit of others. Extreme pervasive and persistent selfishness is a vice” [23]. However, the problem with this statement is that it provides a very poor compass for human enhancement. In fact it offers an extremely flimsy and meager moral guidance. First, we still need to decide whether an action is self-sacrificial (or egocentric). Second, the fact that an action is self-sacrificial obviously does not make it good.

The *specification of betterment problem* does not vanish by making it relative to whatever people find good. Kahane and Savulescu argue that: “For pretty much every objection to biomedical enhancement, it is possible to reply: ‘If what worries you about enhancement is X, then why shouldn’t we try to *enhance X*?’ For example, if you worry that human enhancement will threaten our openness to the unbidden, solidarity with others, or autonomy, then how can you object to biomedical interventions aimed to *increase* people’s openness *to* the unbidden, solidarity, or their autonomous capacities?” [23] Be this as it may, the problem of assuring the connection between HE and betterment (of X) still remains.

Another challenge is that when we enhance humans our value system may change. Hence, what was good yesterday may not be good today. Without a clear, sustainable, and well argued for goal, HE is meaningless. Accordingly, HE contains the sources for limitation (or guidance) in itself. It is not necessary to set “external” limits in terms of naturalness, therapy, or disease. We only need to demand clear, sustainable, obtainable goals that are based on evidence, and not lofty speculations, hypes or weak associations.

#### Objections

In this article I have argued that the traditional resources for setting limits to HE, such as naturalness, therapy, and disease, are not convincing. However, I have found that the lack of specification of betterment inherent in the conception of HE itself provides means to set limits. Human enhancements that specify what becomes better and where adequate evidence that this will happen is provided are good and should be pursued. Others should be limited.

Although the traditional conceptions of disease, therapy, and naturalness may not offer effective limits to human enhancements, alternative or modified versions of such concepts may still do so. To clarify or modify concepts of disease, therapy, or nature (or their contrary concepts health, enhancement, and nature) warrants a separate investigation.

Correspondingly, it may be argued that valid limits are to be found elsewhere, e.g., conceptions of authenticity [34] or in moral psychology. One may argue, for example, that there are moral psychological propensities supporting opposition to enhancement [1] or that various forms of conservativism can do the job [2, 27]. Moreover, limits to HE could also be analyzed in terms of concepts such as “natural purpose” [35], medicalization [87], and others. Strong intuitions and emotions (repugnance) [2, 88–90] also seem to set limits.

The premise of the article is that we need to set limits to HE. As acknowledged, some think that this premise is false. We need HE to improve the world is their argument. To debate the premise is beyond the scope of this article. However, implicitly I have argued that the premise may not be relevant when demanding that HE should only be pursued when it contains a clear, sustainable, and obtainable conception of betterment. There may be no need to limit HE. Demanding clearly formulated, empirically evident, obtainable and sustainable goals of HE may make it self-limiting.

The very endeavor of setting limits may be identified as being “conservative” or “restrictive” [2] in contrast to being “permissive” (or “bioliberal”). Such labelling seems to have more rhetoric than analytical function. By appealing to the clarification of betterment, and endorsing HE that can provide clear, sustainable, obtainable goals for HE, I do not subscribe to any of these labels. They tend to be limiting rather than promoting to a fruitful debate. Moreover, as indicated, the need to set (external) limits may not be as prominent, if we pay more attention to the guiding power of the specification of betterment.

The debate on human enhancement is characterized by licentious speculations where the proponents advocate research and implementation, with caution advised (only if at all) for concerns about safety and justice, and critics see the prospect of HE as an assault on humanity as such. Both tend to rely on unwarranted preconditions (biological determinism) and strong beliefs [91].

It is also important to point out that it is far from obvious that the counterarguments against setting limits to HE hold. On the contrary, I think that several of them are flawed. E.g., the argument against the therapy-enhancement distinction of Guibilini [48] are based on false premises. However, the point in this article has not been to enter into the particular arguments, but rather to illustrate that some of the traditional limit setting resources are not convincing, and to argue that this limit-setting may not be needed, as the goal setting of HE is overly unclear. By learning from the arguments about naturalness, the therapy-enhancement distinction, and disease, we should demand the same clarity and precision of the concept of enhancement as its proponents require from the concepts of nature, therapy, and disease in order to have any limiting or directing function. If it is not clearer what is meant by enhancement than what is meant by nature, therapy, or disease, no more should we pursue the former than it can be limited by the latter.

I agree with Bess that the concept of enhancement is oftentimes played off against concepts like “natural,” “therapeutics,” “normal,” “healthy,” and “disease” as “these concepts form the necessary basis on which most discussions of medicine, technology, and human nature tend to take place” [34]. However, we need to move on in the enhancement debate and more precisely discuss the improvement part of HE: *what exactly is improved and why is it better*?

One may also ask about the limiting power of the specification problem of HE. Will demanding specifications of betterment be sufficient to counter the speculations, hypes, and high hopes of the proponents and to govern HE? One reason why the approach may be effective is that it is easier to specify what is bad than what is good, as there tends to be a basic asymmetry in ethics [92]. After all, it may be easier to define what is disease than what is health and what is treatment than enhancement. Hence, the conceptual and moral challenges may temper the hypes and hopes in a fruitful way – especially if we are able to avoid the confusion of better with more.

## Conclusion

In this article I have investigated the traditional resources for restricting human enhancement, such as the concepts of naturalness, therapy, and disease. These do not seem to do the job. However, the article shows that the concept of enhancement is feeble in defining what is to be enhanced, i.e., goodness. The qualitative *better* is frequently confused with the quantitative *more*. Accordingly, the lack of specification of betterment inherent in the conception of HE itself provides means to restrict its unwarranted proliferation as well as guiding its fruitful implementation. We may therefore not need “external” measures for setting limits in terms of naturalness, therapy, or disease. We only need to demand clear, sustainable, obtainable goals for human enhancement that are based on evidence, and not lofty speculations, hypes, analogies, or weak associations. Human enhancements that specify how humans will become better, and where adequate evidence that this will happen is provided, are good and should be pursued. Others should be restricted.

## Abbreviations

HE: Human enhancement

## Footnotes

1 In fact even authors in the naturalistic camp can claim that the concepts of health and disease are not suitable to delimit the tasks of health care [67].
